# Syphilitic Disease of the Joints

**Published:** 1908-01-18

**Authors:** D'Arcy Power

**Affiliations:** Surgeon to and Lecturer on Surgery at St. Bartholomew's Hospital; Consulting Surgeon to the Bromley Cottage Hospital, Kent


### Hospital Clinics.

### SYPHILITIC DISEASE OF THE JOINTS.

By D'ARCY POWER, F.R.C.S.Eng., Surgeon to and Lecturer on Surgery at St. Bartholomew's Hospital; Consulting Surgeon to the Bromley Cottage Hospital, Kent

An address delivered before the Bromley Medical Society, Kent, on December 10, 1907.

Mr. President and Gentlemen,—It would seem at first sight that, in offering to address you upon syphilitic disease of the joints, I am choosing a subject which is too rare to be of much practical importance in the busy lives you lead as general practitioners of medicine in a district which is rapidly becoming suburban. But syphilis, as you well know, is a far-reaching disease, and its effects are seen not only throughout the lives of patients who have contracted it, but far into the lives of the next generation. The worst effects, too, follow very often upon the mildest attacks because the absence of marked symptoms leads both the patient and his medical man to neglect that prolonged course of treatment by mercury which alone affords a safeguard against the worst ravages of the disease at a later time.

The incidence of syphilis is determined by the irritation to which a part is subjected. The irritation of smoking is followed by mucous patches in the mouth during the earlier stages of syphilis and by chronic glossitis afterwards; persons who have perspired freely during a hot summer or on a walking-tour often suffer from cutaneous syphilides, from which they might otherwise have escaped had their skins been less active; and one of the most troublesome cases of early syphilitic inflammation of the scalp I ever had to treat occurred in a man who worked all day in a tobacco and snuff manufactory.

So, I think, the practice of athletics and the consequent increased use of the joints may in part account for the larger number of syphilitic joint lesions which occur at the present time, as compared with those described by our surgical predecessors, living when billiards were more in vogue than they are to-day, and fashionable youths contented themselves with lounging in the Park or walking in the streets. Be this as it may, reference to the older text-books of surgery shows that syphilitic disease of the joints was hardly recognised, although, as I hope to prove, it occurs in many different forms and at every stage in the disease.

But there is another reason why syphilitic disease of the joints should have been overlooked, and still is too often overlooked. Syphilis is only a factor in joint disease, and, it may be, quite a minor factor. Tuberculous and rheumatic changes give their own colour to the symptoms, and the syphilitic origin passes unknown or unrecognised. This is especially the case in the joint disease of inherited syphilis. A child born of syphilitic parents is treated for inherited syphilis and recovers. The symptoms have never been very severe, and as no signs remain the mother either forgets the early illness or fails to recognise its importance. When the joint symptoms appear later, another practitioner is called in, and he is unable to elicit any history of the previous illness.

Syphilitic inflammation of the joints is also chronic, and we are all too apt to think that every chronic joint disease is necessarily either tuberculous or rheumatic; when once it is satisfactorily labelled it rarely enters into our heads to question the diagnosis. The true reading of the chronic joint affection in many syphilitic children is rather that syphilitic changes in the blood vessels have prepared the tissues for the invasion of micro-organisms. An unusual form of inflammation in a joint which appears to be tuberculous or rheumatic may in reality be a combination of syphilis with rheumatism, tubercle, or gout, when the arthritis is chronic; whilst if it be more acute and suppurates, the infective agents may be staphylococci, pneumococci, or streptococci. The difference in the clinical symptoms and course is due to the preponderance of one or other of the infective agents. It is quite possible that in these complex cases the timely administration of mercury may so far strengthen the inherent weakness of the tissues, by arresting the syphilitic taint, as to prevent them from becoming a nidus for other micro-organisms. It is worth while, therefore, to keep such patients under the influence of mercury for long periods of time, because the presence of even a small quantity of mercury in the tissues is sufficient to inhibit the action of the syphilitic virus.

Syphilitic affections of the joints are clearly subdivided for clinical purposes into inflammations occurring in the course of acquired and of inherited syphilis. But nature knows no such arbitrary classifications, and the pathological processes are identical in each case. The different forms may be conveniently tabulated in the following manner:—

| | | | |
|---|---|---|---|
| Acquired | Arthralgia | | |
| | Synovitis | Serous | Painless<br>Painful |
| | | Gummatous | |
| | Arthritis | Ulcerating or Virchow's joints<br>Sclerosing or Charcot's joints | |
| Inherited | Synovitis | Suppurative<br>Symmetrical or Clutton's joints.<br>Gummatous | |
| | Arthritis | Ulcerating or Von Gies' joints. | |

It will thus be seen that the joint affections occurring as a result of acquired syphilis are of several different kinds, and may manifest themselves at any period after the disease has been contracted.

### A. Acquired Syphilis.

1. *Arthralgia, or Syphilitic Neuralgia of the Joints.*

The first affection of the joints to show itself as a result of acquired syphilis is a persistent neuralgia, to which the name "arthralgia" is given for purposes of convenience. The pain is usually felt during the first few months after the infection, and is coincident, therefore, with mucous patches in the mouth and the transient skin eruptions of early syphilis. It is sometimes felt before the hard sore has appeared. Women complain of the pain more frequently than men, and it is felt in the shoulder and knees more often than in the elbows, wrists, or ankles; complaint is rarely made of the hips. The pain is usually limited to a single joint, and examination shows no cause for it, as there is neither heat, swelling, nor redness, nor does pressure materially increase the pain. The patient is thought, therefore, to complain unnecessarily, or the discomfort is attributed to some form of rheumatism. No treatment is employed, or a course of salicylates is ordered, but without giving any relief. Left to itself the pain disappears, like all the earlier manifestations of syphilis, after a period varying from a few weeks to a few months.

A little skilful cross-examination will give a clue to the cause of the arthralgia, and this in turn will lead to a correct diagnosis and proper treatment; so that the patient may be saved many weeks of somewhat wearing pain and the medical man will obtain a corresponding amount of credit for his treatment.

The first point which distinguishes arthralgia from rheumatism is the fact that the pain is worse in the morning and wears off towards evening. This fact alone should awaken the suspicions of the medical attendant, for pains without external manifestations and worse at night have long been known, in the form of bone pains and headaches, as characteristic of early syphilis. A careful examination of the painful joint, too, shows that the neuralgia is not wholly or even chiefly connected with the joint, but is rather a general tenderness of the various structures in the neighbourhood of the part about which complaint is made: the muscles, ligaments, tendons, aponeuroses and periosteum all seem to be tender.

The pathology of the condition is unknown, though we may guess, on the analogy of other forms of infective arthritis, that it is due to toxins produced by the special form of spirochæte which causes syphilis. Indeed, it is remarkable that neuralgia was much commoner when syphilis was more virulent than it is at present. For the European nations are now more or less immunised to the disease. The older writers, therefore, make frequent reference to the painfulness of syphilis, whilst we are rather accustomed to think of the disease as comparatively free from pain.

Arthralgia is cured very quickly by the administration of mercury; indeed, the condition is so amenable to this drug that the pain is relieved by the local application of Scott's dressing, under the mistaken idea that the joint is affected with rheumatism or tubercle, or by the first doses of mercury given for the relief of a mucous patch or secondary syphilide. The treatment, therefore, is very simple. A course of mercury, whether in the form of grey powder or corrosive sublimate, given by the mouth, or by intramuscular injection of Lambkin's cream or grey oil, will speedily arrest the pain and remove any local tenderness which may be present.

### 2. *Synovitis.*

The synovial membrane of the joints is affected in several ways by the syphilitic poison. In the simplest form there is a painless swelling of the joint without any rise of temperature. The knees are more often involved than the shoulders, and the ankles more frequently than the wrists. A single joint may be swollen, but the hydrarthrosis is often symmetrical.

(*a*) *Serous Synovitis or Hydrarthrosis.*—In the slightest forms the swelling passes unnoticed by the patient because it is so painless. But he may complain of a weakness of the knees in walking, or the medical man may discover the condition whilst he is examining the body for other signs of syphilis. Left to itself the swelling soon subsides even without treatment, though recurrent attacks are not very uncommon. Under the action of mercury, whether administered internally or by local application, the swelling soon subsides, and there is no evidence to show that the joint is permanently injured by the swelling. The swelling is so often associated with other signs of early syphilis that it is not likely to be mistaken for anything else, and the diagnosis and treatment of the condition are consequently easy.

A rarer form of serous synovitis due to syphilis is likely to be mistaken for rheumatism. It occurs during the earlier period of the disease, and is characterised by a sudden and painful swelling of one or more of the larger joints, with some reddening of the skin over the swollen part. The knees, ankles, wrists, and elbows are most frequently affected, and the condition is not relieved by salicylates, though the pain and swelling rapidly disappear when mercury is given. The diagnosis is made by knowing of the co-existence of syphilis, by the absence of fever, and by the limitation of the symptoms to the larger joints, for in a case of subacute polyarticular rheumatism of like degree some of the smaller joints would certainly be affected. The prognosis is not quite so good as in the simple synovitis just described, because the affected joints afterwards creak as though they had been the seat of osteoarthritis. It is important, therefore, to recognise and cure the inflammation as quickly as possible, and not to allow it to continue under the idea that it is some intractable form of rheumatism. Skilfully applied massage is very useful in these cases of synovitis.

(*b*) *Gummatous Synovitis.*—The later manifestations of joint disease in acquired syphilis are of great interest, and the most common perhaps is a gummatous synovitis, which is very likely to be mistaken for tuberculous disease. The gummatous inflammation affects any of the larger joints, and I have seen it most often in the knee, the ankle, the elbow, and the shoulder. It has always been present for a long time before the patient complains of the joint, because it is painless and does not impede movement for a considerable period. The inflammation is generally confined to a single joint, and in the case of the knee the patient limps into the room, saying that his knee is weak and swollen and that he can no longer walk as he used to do. You look at the joint. The first impression is that it is a large tuberculous synovitis, and the thought passes through your mind, "How in the world has the man walked about with such a knee, it must be horribly painful?" It is enlarged and oval without any reddening of the skin, just like an ordinary case of tuberculous tumor albus. Closer examination shows that the swelling is not quite so oval as in the tuberculous form, because the muscles of the thigh are less wasted, whilst the swelling itself is less uniform. Besides, the patient assures you that he has very little pain, and he proves it by flexing and extending the joint to show that the movements are really painless. Still closer examination shows that the thickening is by no means so uniform as it is in cases of tuberculous synovitis. Harder nodules are to be felt in some parts of the synovial membrane, whilst in other parts the synovial membrane is not more perceptible than it is in health. A line of thickening often runs across the joint just above the upper border of the patella. The tissues surrounding the joint are thickened, and the periosteum of the femur is also thickened for some distance up the shaft. The movements of the joint, too, are perfectly smooth, and there is no undue amount of lateral motion. Yet in spite of all these minor differences which distinguish gummatous from tuberculous synovitis, and should be sufficient to put one on one's guard as to the accuracy of the diagnosis, mistakes are constantly made; having labelled the joint tuberculous, we are surprised that it does not improve on anti-tuberculous treatment.

Fixation of the joint with extension in the manner adopted in tuberculous synovitis is nearly always tried first in gummatous synovitis, and without effect except perhaps to reduce the size of the joint a little owing to the rest which is afforded, nor does the patient obtain any benefit from the cod-liver oil which is ordered for him. Indeed, throughout his illness a patient with gummatous synovitis remains otherwise in good health, and presents a very different picture from the patient with tuberculous synovitis. He often has no characteristic syphilitic signs, nor does he present any rise of temperature or other evidences of tuberculous infection.

Under the influence of mercury, combined with a short course of iodide of potassium, the joint improves marvellously in a very short space of time. It is evident that the mercury is the chief agent in causing this improvement, because it takes place to a certain extent when a Scott's dressing is applied to the joint to "take down the inflammation." Indeed, I have sometimes wondered whether Scott's dressing does not owe its reputation in chronic disease of the joints to errors in diagnosis. A century ago John Scott (1798-1846) was surgeon to the London Hospital, and his father James Scott practised in the town of Bromley, Kent. Syphilis was very much more common in England during the eighteenth and nineteenth centuries than it is at the present time, owing to the drinking habits of all classes. Syphilitic synovitis, therefore, was more common than it is at the present time, though tubercle was certainly not less common when ladies were accustomed to spend the evenings, at Vauxhall and Cremorne, in very low dresses. Both conditions gave rise to a white swelling of the knee, and as one was not distinguished from the other, Scott's plaster, containing a camphorated mercurial compound, soon gained a great reputation for curing cases of tumor albus. This reputation has not been maintained since we have been able to diagnose between tubercle and syphilis—at least, I have never satisfied myself that a really tuberculous joint has been the better for the application of a Scott's dressing.

Dissection of such a joint shows that the gummatous inflammation has affected the outer layers of the synovial membrane and the surrounding fibrous tissues. The endothelial lining is shiny, and the articular cartilages are smooth and healthy. The pathological condition, therefore, is a perisynovitis rather than a true synovial inflammation.

The prognosis in the simpler cases of perisynovial gummatous inflammation is good, for under the influence of mercury given by the mouth or by injection the swelling rapidly subsides, and the patient makes a good recovery with a useful joint, though it sometimes feels weak owing to relaxation of the ligaments from the prolonged stretching which they have undergone as a result of the long-continued swelling.

### 3. *Arthritis.*

The next two conditions of joint disease result rather from the effects of acquired syphilis than from the disease itself.

*Chondro-arthritis* is a chronic and painful affection of the joints which occurs very many years after the disease has been acquired. It is really more common than the published cases would lead one to suppose, because most of the cases are thought to be examples of senile tubercle or of osteoarthritis, the more so because the history of syphilis is so lost in the mists of antiquity that it is difficult for the patient to recall the details of an attack, especially when there is reason to believe that the disease ran a very mild course. I have had at least two cases of chondro-arthritis under my care this year, and the following details of one of them will be sufficient to show the general course taken by the disease. I select it because it ended in amputation, and we thus had an opportunity of examining the joint.

A night-porter, aged 38, was admitted into St. Bartholomew's Hospital under my care on January 15, 1907, complaining of pain and swelling in the right knee. He said that he had been quite well until November, 1906, when he was suddenly seized with shivering and pain in his knuckles, fingers, wrists, knees, and ankles. The joints swelled and were hot, but on the third day the pain and swelling left them all, except the right knee, which got worse instead of better. An Austrian by birth, he had travelled extensively, and had suffered from venereal disease in his youth, but there were no signs of tabes. On admission he was found to be a healthy-looking man, whose right leg and thigh were swollen, the tissues pitting on pressure. The leg was kept extended, and any movement of the knee caused great pain. The right knee was swollen and the tissues surrounding it were much thickened.

The pain continued unabated until, on January 24, I opened the joint and let out several ounces of blood-stained synovial fluid. The wound never healed satisfactorily, and the joint afterwards suppurated. I amputated the thigh in the lower third on April 8, and on April 19 there was such severe hæmorrhage from the stump that I tied the femoral artery in Scarpa's triangle. The hæmorrhage was a general oozing from both flaps, and although ten days had elapsed since the operation no repair had taken place. The patient made a satisfactory recovery, and left the hospital on May 21 with a soundly healed stump.

An examination of the diseased joint showed that the synovial membrane was œdematous and greatly thickened, for it was five-eighths of an inch thick at the point where it was reflected upon the side of the joint. The villous processes were enlarged into lobules, and did not present the appearances usually seen in cases of osteoarthritis. The articular cartilages were extensively destroyed, leaving the underlying bone exposed and rarefied. The remaining cartilages were succulent, blood-stained, and pitted by crescentic erosions, and the bone was marked in parts by a linear striation, though it was nowhere eburnated. A microscopic examination of the synovial membrane showed the arteries to be thickened in the manner often seen in syphilis, whilst the synovial membrane contained softened and caseating patches, which were not tuberculous. The cartilages had fibrillated parallel with the surface of the bone, and not vertically, as is usual in joints affected with osteoarthritis.

The patient was treated with mercury administered both by the mouth and by injections, as well as with doses of iodide of potassium, but he obtained no relief from either of these drugs until after his thigh had been amputated.

These cases of ulcerating gummatous arthritis occurring late in the course of acquired syphilis form an important group, which stands in need of further investigation. They seem to be the result of a mixed infection acting upon tissues which have long been diseased by the syphilitic virus. In the case I have just quoted the patient appeared to have suffered from syphilis. He was a night-porter, who had much stair-climbing in the course of his work, and he thus used his knee-joints excessively. He had an attack of influenza with joint complications. I opened his knee, and further added to the infective micro-organisms by failing to secure union by first intention. The chronic arthritis was converted into a suppurative inflammation, upon which were impressed many of the characteristics of its syphilitic origin. The absolute failure of union after amputation and the occurrence of secondary hæmorrhage were noteworthy points, for they very rarely occur nowadays; repair took place without further trouble as soon as I ordered him a drachm of the liquor hyd. perchlor. with 20 grains of potassium iodide three times a day.

### Charcot's Joints.

I need hardly say much about Charcot's joints as one of the latest manifestations of acquired syphilis, for they are well known to everyone, and their association with locomotor ataxy makes them easy to recognise. The disease may be limited to one of the large joints, and it is then most common in the knee and the hip; but in many cases several joints are affected simultaneously, and the smaller articulations, like those of the toes and thumbs, suffer equally with the ankles, wrists, and elbows. The rapid course, the destruction of the bone, the absence of pain, and the production of a "flail joint," are sufficient to distinguish a case of Charcot's disease from the more usual condition of osteoarthritis, especially if the signs of tabes be noticed at the same time. But it happens in a certain proportion of cases that the tabetic symptoms follow instead of precede the joint affection. Palliative measures are usually the only method of treatment in these troublesome cases, because the inflammation is not limited to a single joint; but in a case which was recently under my care I amputated, at the urgent request of the patient, after I had satisfied myself by prolonged delay that the knee alone was affected. The details of the case were briefly as follows:—

A cook, aged 45, was admitted into St. Bartholomew's Hospital under my care on July 18, 1907, on account of a useless and painful left leg. She said that she was the widow of a soldier who had served twelve years in India, and who died of phthisis four years after marriage. She had borne him one child, who died of convulsions whilst teething, but she had never miscarried. Her left knee suddenly became swollen in 1905. It was not painful, and for a few months afterwards she could get about. The leg gave way under her one day, and the knee was useless ever afterwards. She had suffered from lightning pains in her legs for ten or twelve years, and for six months before her admission to the hospital she had only partial control over her bladder. An examination of the left knee showed that it was greatly swollen; the joint contained free fluid; it grated on movement, and was distinctly "flail-like"; the articular cartilages were lipped; osteophytes were present, and the patella was displaced inwards. All the other joints in the body seemed to be healthy. As I had watched the patient for more than a year, and satisfied myself that the left knee was alone affected, I yielded to her urgent request and amputated through the lower third of the thigh on July 25. The flaps healed by first intention, and she went to the Swanley Convalescent Home on August 14.

### B. Inherited Syphilis.

Joint disease due to inherited syphilis occurs in several forms, which are easily distinguished, but there are others in which it is extremely difficult to say how much of the disease is due to syphilis and how much to other infective micro-organisms, notably tubercle and pneumococcus.

### 1. Synovitis.

(*a*) *Suppurative Synovitis.*—If the inflammations of joints due to inherited syphilis be taken according to the age at which they show themselves, suppurative synovitis certainly comes first. In its simplest form it begins in the joints of marasmic infants affected with syphilis during the first few weeks of life. Sometimes it attacks only a single large joint, but more often two or three are affected. The capsule soon yields, and extensive diffuse abscesses are formed, which may open spontaneously. Although the baby presents clear evidence of syphilis, there is very little doubt that the suppuration is due to secondary infection of the joint with some pyogenic organism, the staphylococcus and pneumococcus being generally the offending agents. The inflammation of the joint is sometimes secondary to syphilitic disease of the epiphyseal line, when an abscess finds its way into the joint either by a circuitous route or by direct penetration of the epiphysis.

The treatment in these cases of suppurative arthritis consists of laying the joint open freely and draining it for some days whilst the patient is treated with mercury. The prognosis is not nearly so bad as might be expected, for in several babies treated in this manner I have seen perfectly useful joints some years afterwards.

(b) *Symmetrical Serous Synovitis.*—This condition is now well known, as it affects children between the eighth and the fifteenth years. The knees are most often affected, and the inflammation is usually symmetrical, one joint being affected before the other. The wrists and ankles are occasionally affected. The inflammation takes the form of a very chronic serous synovitis, which is usually painless. The joint is only partially filled with fluid, so that it never feels tense; it is not hot; there is no redness of the skin, no starting pain at night, no wasting of the muscles, and no creaking. Movement of the joint does not cause pain, though the child may complain of the joint feeling weak. Examination of the joint shows that the swelling is due in some cases to a simple synovial effusion, the synovial membrane remaining unthickened; in other cases nodules can be felt in the synovial membrane, irregularly distributed and varying in size; whilst in a third form there is comparatively little synovial effusion, though the membrane is very greatly thickened. The treatment consists of the administration of mercury, and the greater the thickening of the synovial membrane the better, in my experience, is the prognosis; the most unsatisfactory cases I have had to treat have been those in which there was very little thickening with a large amount of fluid. The improvement may sometimes be hastened by giving iodide of potassium in combination with mercury.

(c) *Gummatous Synovitis.*—Syphilitic inflammation of the joints due to inherited disease occurs either as a gummatous synovitis or as a true arthritis, in which all the tissues of the joint are involved. Gummatous synovitis is extremely likely to be mistaken for tuberculous disease of the joint, but its more chronic course—the marked though slight evidence of syphilis in other parts of the body and the smaller amount of pain—will generally serve to distinguish the two forms . f disease, unless, as sometimes happens, tubercle bacilli are active in tissues which have already been the seat of syphilitic inflammation. The prognosis of gummatous synovitis is good, and the following case illustrates very well the course run as well as the treatment to be adopted for its cure:—

A boy, aged 14, came under my care on the last day of January with an inflammation of the right knee. He had diphtheria in September, and about a month afterwards sores appeared on his body. He was noticed to be walking lamely about the end of December. The patient's complexion was muddy, and scattered over his limbs and scalp were patches of superficial ulceration, covered with thick scales or with black and raised crusts. The voice was husky but his teeth were healthy, and there was no evidence of keratitis or iritis, either recent or remote. The glandulæ concatenatæ at the posterior borders of the sterno-mastoid muscles were slightly enlarged on both sides. The right knee was affected with synovitis, and there was some increase of synovial fluid in the left knee. The synovial membrane in both joints seemed to be thickened, especially at the sides. The patient felt a little throbbing pain, but unless the knee were moved it was never severe, and he had not been awakened by starting pains at night.

The boy was brought by his mother, who said that a younger brother had had one ankle-joint excised for a similar condition, which had presumably been mistaken for a tuberculous arthritis; but she presented such obvious signs of tertiary syphilis that the boy she brought to me was at once taken into the hospital and put upon a course of grey powder. In ten days' time his complexion had cleared and he was less husky. There was also a smaller quantity of fluid in his right knee, and none in the left. The improvement continued until March 7, when the patient was allowed to go about with his knee in a plaster-of-Paris case, and he was discharged from the hospital. He returned on March 26 without the case, and complaining of much pain with increased swelling in both knees. Both joints were fixed with plaster-of-Paris bandages; but he again returned on April 2, with more swelling in the knees and some synovitis of both elbows. He then confessed that he had not taken his powders for a fortnight. The boy was made to understand that medicine was necessary for his cure, and he was ordered to continue the grey powders. His elbows were less swollen and painful on April 9, though his knees still remained enlarged. He was therefore given half-drachm doses of the solution of perchloride of mercury, with five grains of iodide of potassium, three times a day. He returned a week afterwards, saying that his elbows were well, his knees better, and that he had suffered no pain since he began the new medicine. He increased in weight from 64½ lb. on April 16 to 71½ lb. on April 30, and he has since remained well and at work.

### 3. Chondro-Arthritis.

The worst form of syphilitic arthritis is fortunately the rarest, for it does not respond to the ordinary antisyphilitic remedies and it is practically incurable. It is that form of inherited syphilis which is seen as one of the later manifestations about the time of puberty. The bones and joints are extensively affected by a rarefying osteitis, with a deposit of caseating material in the cancellous tissues. A similar process takes place in the articular cartilages, and leads to the formation of irregular pits and grooves. The condition may be preceded by transient attacks of synovitis with evidence of chronic inflammation of the long bones. There are usually many other manifestations of syphilis, and there are often marked intraocular lesions. The condition is analogous to the chondro-arthritis of acquired syphilis, but in the cases which I have seen in children the gummatous periostitis and osteitis are marked features, whilst in adults the inflammatory changes are more strictly limited to the tissues of the joint. Chondro-arthritis in children is very likely to be mistaken for tuberculous disease. But it is distinguished by the slower course run by chondro-arthritis, by the slighter tendency to fungation and suppuration, and by their association with other unmistakable signs of inherited syphilis.

The result of treatment in advanced cases is most unsatisfactory. Mercurial vapour baths perhaps afford some chance of improvement, with the inunction of ten-per-cent. solution of oleate of mercury at the affected joints, whilst Lambkin's cream is injected into the muscles of the buttock once a week.

There are a few other forms in which syphilis affects the joints, as in the case of syphilitic spine and syphilitic dactylitis; but these joint troubles really begin in the bone, and only spread to the joints secondarily, so that they do not properly come under our notice this evening. I think I have fulfilled my promise, and have shown you that syphilitic diseases of the joints are neither few nor unimportant. Much can be done by treatment for some of the forms, little for others, nothing, unfortunately, for a few. But, as in every other joint disease, early recognition gives the best results if appropriate treatment be ordered; and it must be constantly in our mind that syphilitic disease of the joints is mistaken for rheumatism and osteoarthritis in the old, for acute rheumatism and for tubercle in the young.

